# Low‐Frequency Ultrasound Sensitive Piezo1 Channels Regulate Keloid‐Related Characteristics of Fibroblasts

**DOI:** 10.1002/advs.202305489

**Published:** 2024-02-04

**Authors:** Zixi Jiang, Ziyan Chen, Yantao Xu, Hui Li, Yixin Li, Lanyuan Peng, Han Shan, Xin Liu, Huayi Wu, Lisha Wu, Dan Jian, Juan Su, Xiang Chen, Zeyu Chen, Shuang Zhao

**Affiliations:** ^1^ Department of Dermatology Xiangya Hospital Central South University Changsha 410008 China; ^2^ Furong Laboratory (Precision Medicine) Changsha 410008 China; ^3^ National Engineering Research Center of Personalized Diagnostic and Therapeutic Technology Xiangya Hospital Central South University Changsha 410008 China; ^4^ Hunan Engineering Research Center of Skin Health and Disease Xiangya Hospital Central South University Changsha 410008 China; ^5^ Hunan Key Laboratory of Skin Cancer and Psoriasis Xiangya Hospital Central South University Changsha 410008 China; ^6^ National Clinical Research Center of Geriatric Disorders Xiangya Hospital Central South University Changsha 410008 China; ^7^ School of Mechanical and Electrical Engineering Central South University Changsha 410083 China; ^8^ Department of Dermatology Xijing Hospital The Fourth Military Medical University Xi'an Shaanxi 710000 China

**Keywords:** fibroblast, keloid, low‐frequency ultrasound, Piezo1

## Abstract

Keloids are benign fibroproliferative tumors that severely diminish the quality of life due to discomfort, dysfunction, and disfigurement. Recently, ultrasound technology as a noninvasive adjuvant therapy is developed to optimize treatment protocols. However, the biophysical mechanisms have not yet been fully elucidated. Here, it is proposed that piezo‐type mechanosensitive ion channel component 1 (Piezo1) plays an important role in low‐frequency sonophoresis (LFS) induced mechanical transduction pathways that trigger downstream cellular signaling processes. It is demonstrated that patient‐derived primary keloid fibroblasts (PKF), NIH 3T3, and HFF‐1 cell migration are inhibited, and PKF apoptosis is significantly increased by LFS stimulation. And the effects of LFS is diminished by the application of GsMTx‐4, the selective inhibitor of Piezo1, and the knockdown of Piezo1. More importantly, the effects of LFS can be imitated by Yoda1, an agonist of Piezo1 channels. Establishing a patient‐derived xenograft keloid implantation mouse model further verified these results, as LFS significantly decreased the volume and weight of the keloids. Moreover, blocking the Piezo1 channel impaired the effectiveness of LFS treatment. These results suggest that LFS inhibits the malignant characteristics of keloids by activating the Piezo1 channel, thus providing a theoretical basis for improving the clinical treatment of keloids.

## Graphical abstract

Low‐frequency sonophoresis inhibits the migration of patient‐derived primary keloid fibroblasts (PKF), NIH 3T3, and HFF‐1 cells and increases PKF apoptosis via activating Piezo1 channel. Establishing a patient‐derived xenograft keloid implantation mouse model further verifies these results, as LFS significantly decreases the volume and weight of the keloids. Moreover, blocking the Piezo1 channel impairs the effectiveness of LFS treatment.

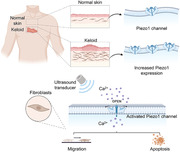

## 1 Introduction

Keloids are benign fibroproliferative tumors characterized by excessive dermal fibroblast proliferation and extracellular matrix deposition, affecting 4.5–16% of the human population.^[^ ^1^, ^2^, ^3^ ^]^ While keloids are often considered a cosmetic issue, they adversely affect a patient‘s quality of life by causing lesional and perilesional pruritus and pain and affecting the normal mobility of the lesion area.^[^ ^4^ ^]^ Moreover, keloids pose a heavy financial burden on healthcare, with associated costs amounting to billions of dollars annually.^[^ ^5^ ^]^ Surgery is the primary treatment for various types of keloids but is also a well‐known trigger.^[^ ^6^ ^]^ Due to the high prevalence of keloids arising from wounds (5–15%) and the substantial recurrence rates (70–100%) observed in keloids previously treated through surgical excision,^[^ ^2^, ^3^, ^7^ ^]^ a significant number of patients with keloids exhibit apprehension toward surgical interventions.

According to this, significant advancements have been made in the development of innovative non‐surgical technologies including laser, intense pulsed light, radiofrequency, and ultrasound.^[^ ^8^, ^9^ ^]^ Ultrasound is a versatile medical technology with a broad spectrum of clinical applications, ranging from diagnostic imaging to therapeutic interventions,^[^ ^10^, ^11^, ^12^, ^13^ ^]^ owing to its inherent safety and minimal invasiveness. The ultrasound mechanism is based on the generation of acoustic waves, cavitation, and thermal effects.^[^ ^14^, ^15^ ^]^ Ultrasound technology is often combined with a fractional laser or microplasma radiofrequency technology to facilitate the penetration of anti‐scarring drugs.^[^ ^16^, ^17^ ^]^ Ultrasound can help enhance the penetration of drugs into deeper tissue layers rather than directly exhibiting treatment efficacy on keloids. When subjected to ultrasound treatment, the application of high‐frequency vibrations and propagation of ultrasound waves result in the generation of shear and acoustic radiation forces acting on the cells within the tissue. Although the primary mechanisms responsible for enhanced skin permeability by ultrasound, commonly known as sonophoresis, are generally accepted as the main contributors, including acoustic cavitation and mechanical benefits,^[^ ^18^ ^]^ the biophysical mechanisms are not yet fully understood. The efficacy of ultrasound as a standard treatment for keloids remains unclear.

We analyzed public single‐cell data and found that the Piezo1 channel was significantly overexpressed in keloid tissues compared to normal scars,^[^ ^19^ ^]^ indicating that Piezo1 plays a potential role in keloids. Piezo1 serves as a mechanosensitive ion channel that plays a crucial role in sensing mechanical stimuli in cells. It directly converts mechanical forces such as static pressure, shear stress, and membrane stretch into intracellular biological signals. Previous studies have demonstrated that Piezo1 plays an important role in the mechanotransduction of ultrasound‐stimulated responses in dental stem cells,^[^ ^20^ ^]^ osteoblastic cells,^[^ ^21^ ^]^ pancreatic cancer cells,^[^ ^22^ ^]^ and neuronal cell lines.^[^ ^23^ ^]^ However, the biophysical mechanisms of fibroblast function have not yet been fully elucidated.

Here, the effects and biophysical mechanisms of low‐frequency sonophoresis (LFS) stimulation in fibroblasts were investigated. To achieve drug penetration, a customized ultrasound transducer with 110 kHz frequency was used instead of a high‐frequency ultrasound (≥0.5 MHz) used in previous studies. LFS is more effective than high‐frequency sonophoresis for enhancing skin permeability.^[^ ^24^ ^]^ Our results showed that LFS activated the Piezo1 channel, leading to an influx of Ca^2+^, an essential secondary messenger in cellular signaling. LFS inhibits the migration of fibroblasts and increases the apoptosis of patient‐derived primary keloid fibroblasts in a Piezo1‐regulated manner. Moreover, LFS partially hindered keloid growth in vivo by activating the Piezo1 channels.

## 2 Results

### 2.1 Increased Expression of Piezo1 in Keloids

**Figure 1:**
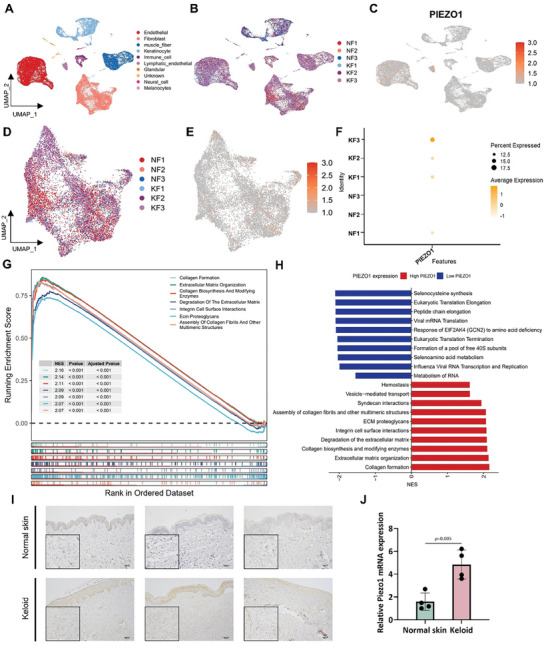
Higher expression of Piezo1 in keloids compared with normal scar and normal skin. A) Unbiased clustering reveals 10 cellular clusters. Clusters are distinguished by different colors. The general identity of each cell cluster is shown on the right. B) The sources of cells in cellular clusters. NS: normal scar; KL: keloids. C) Feature plots of the expression distribution for Piezo1 in each cellular cluster. D) The sources of fibroblasts. NF: normal scar fibroblasts; KF: keloid fibroblasts. E, F) Feature plots of the expression distribution for Piezo1 in fibroblast cluster. G) GSEA enrichment plots for representative signaling pathways upregulated in KF compared to NF. H) GO Biological Process enrichment analysis of differentially expressed genes between KF and NF. I) Immunohistostaining of Piezo1 in the dermis of keloids and normal skin (n = 3). J) Relative mRNA expression level of Piezo1 in keloids and normal skins (n = 3). *p* Value was calculated using Student's *t*‐test.

Previous studies have reported overexpression of Piezo1 in myofibroblasts of human and rat hypertrophic scar (HS) tissues, and Piezo1 plays an important role in the process of mechanical stretch‐promoted HS formation.^[^ ^25^ ^]^ To investigate whether the expression of Piezo1 increased in keloids, we compared the expression levels of Piezo1 in keloids and normal scars based on single‐cell RNA‐seq (scRNA‐seq) data previously published by Deng et al.^[^ ^19^ ^]^ As a mechanosensitive ion channel, Piezo1 was more frequently expressed in endothelial and lymphatic endothelial cells (**Figure** **1**A–C). Furthermore, Piezo1 was significantly increased in keloid fibroblasts compared with normal fibroblasts (Figure 1D–F). Gene set enrichment analysis (GSEA) and gene ontology (GO) analyses suggested that collagen biosynthesis and modifying enzymes, collagen formation, and extracellular matrix organization‐related pathways were enriched in keloid fibroblasts (Figure 1G,H). To validate the findings identified by scRNA‐seq, histological analysis was conducted on keloid and normal skin sections. Compared to normal skin, immunohistochemical staining showed higher Piezo1 expression in the dermis of keloids (Figure 1I). The relative Piezo1 mRNA expression level was consistently higher in keloid lesions than in normal perilesional skin (Figure 1J).

### 2.2 Low‐Frequency Sonophoresis System

**Figure 2:**
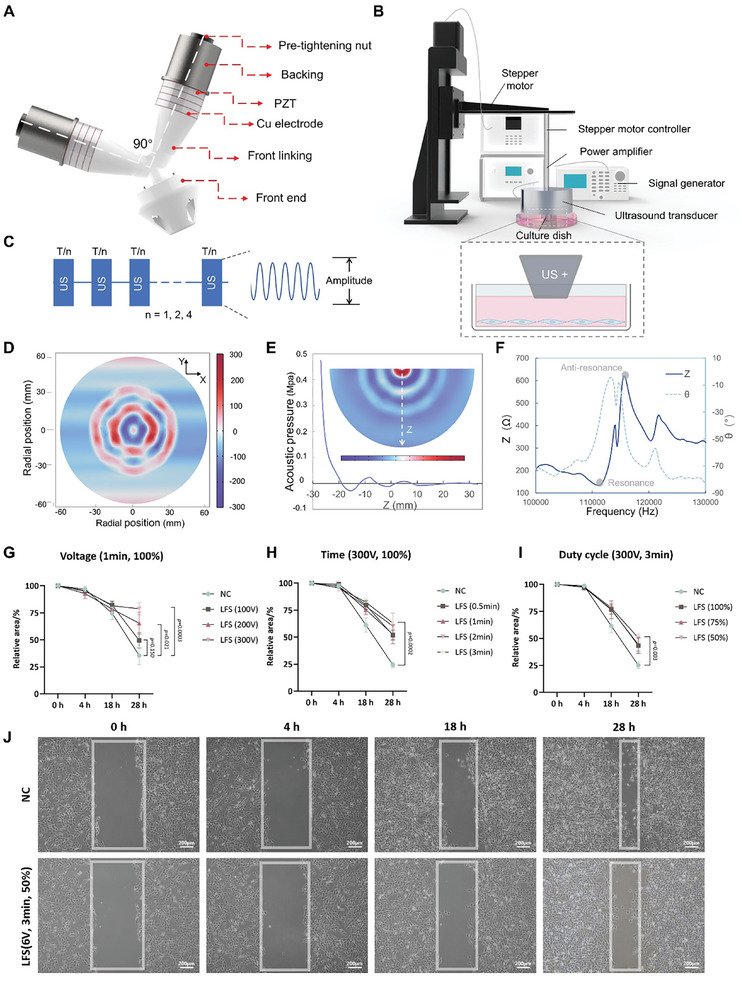
LFS stimulation system. A) Schematic diagram depicting the structure of the LFS transducer. B) Schematic diagram of the experimental setup for the LFS stimulation of fibroblast cells. C) Schematic illustration of the LFS stimulation parameters. D) Finite element method (FEM) simulation of LFS acoustic pressure contact with the water surface. E) FEM simulation of LFS acoustic pressure along the depth direction Z. Same bar. F) Impedance and phase angle spectra of the LFS transducer. The resonant and antiresonant frequencies (111 and 116 kHz) are labeled with shaded circles. G) The effects of LFS voltage in the migration of NIH 3T3 cells (time: 1 min, duty cycle: 100%, n = 4). H) The effects of LFS time in the migration of NIH 3T3 cells (voltage: 300 Vpp, duty cycle: 100%, n = 6). I) The effects of LFS duty cycle in the migration of NIH 3T3 cells (voltage: 300 Vpp, time duration: 3 min, n = 4). *p* Values were calculated using Student's *t*‐test. J) Randomly selected images of the gap of NIH 3T3 cells at 0, 4, 18, and 28 h after LFS. Scale bar, 200 µm.

To realize LFS, we designed a dual‐probe ultrasound transducer. The transducer consisted of two Langevin transducers at an angle of 90°, which could generate a deeper and stronger radial acoustic field than a single transducer (Figure S1, Supporting Information). Each transducer had a front end, front linking, Cu electrode, PZT sheets, backing, and pre‐tightening nuts (**Figure** **2**A). The Langevin transducer transmitted ultrasound through the front end. The utilization of the backing enhances the forward radiation power of the ultrasound while ensuring direct contact with air, thereby facilitating efficient heat dissipation. Under sinusoidal voltage excitation, a Langevin transducer can produce mechanical vibrations and acoustic radiation. During the experiment, the surface of the transducer was immersed in the culture dish medium and controlled using a stepper motor. The signal generator generated a sinusoidal voltage using a power amplifier to excite the ultrasound transducer (Figure 2B,C). The acoustic field generated by the LFS is simulated using a finite element model (FEM). At the excitation voltage of 200 Vpp, the maximum acoustic pressure was ≈0.5 MPa. The surface acoustic pressure was alternating between positive and negative and decayed along the depth direction (Figure 2D,E).

The resonant frequency and ultrasound intensity are two important parameters of an ultrasound system. An impedance experiment was conducted to determine the resonant frequency of the LFS. An impedance analyzer (ZX70A, Changzhou Zhixin Precision Electronics Co., Ltd., China) was used for the impedance test, and the measured resonant frequency was 111 kHz (Figure 2F). Ultrasound intensity was measured using a colorimetric method.^[^ ^26^ ^]^ The measured power intensity was ≈3 W cm^−2^, indicating the safety and suitability of the LFS system for in vivo experiments.

It has been recognized that classic keloids spread aggressively, rarely resolve spontaneously, and, at the histologic level, exhibit an abundance of keloidal collagen.^[^ ^27^ ^]^ Therefore, an ideal treatment for keloids should inhibit the migration, viability, and collagen synthesis of fibroblasts. To investigate whether LFS stimulation influenced the migration of fibroblasts, we performed scratch wound healing assays with different LFS parameters: excitation voltage, duration, and duty cycle. The results showed that LFS inhibited the migration of NIH 3T3 cells and exhibited optimal migration inhibition efficacy at a voltage of 300 Vpp, duration of 3 min, and a duty cycle of 50% (Figure 2G–J; Figure S2, Supporting Information). Thus, we selected these parameters as the optimal technical conditions for the following applications: To further examine whether LFS influenced fibroblast viability and collagen synthesis, we performed flow cytometry to detect apoptosis, quantitative reverse transcription PCR (qRT‐PCR), and immunofluorescence staining. Although we did not observe an apoptosis inhibitory effect of LFS on NIH 3T3 cells (Figure S3, Supporting Information), the collagen type I (*COL1*) mRNA level was relatively lower (Figure S4A, Supporting Information, *p* = 0.338), and collagen type III (*COL3*) mRNA levels were significantly lower (Figure S4B, *p ≤* 0.0001) after LFS stimulation, and the fluorescence intensity of *COL1* and *COL3* in LFS was significantly lower in the LFS group (Figure S4C,D, Supporting Information).

### 2.3 The Expression of Piezo1 in Fibroblasts

**Figure 3:**
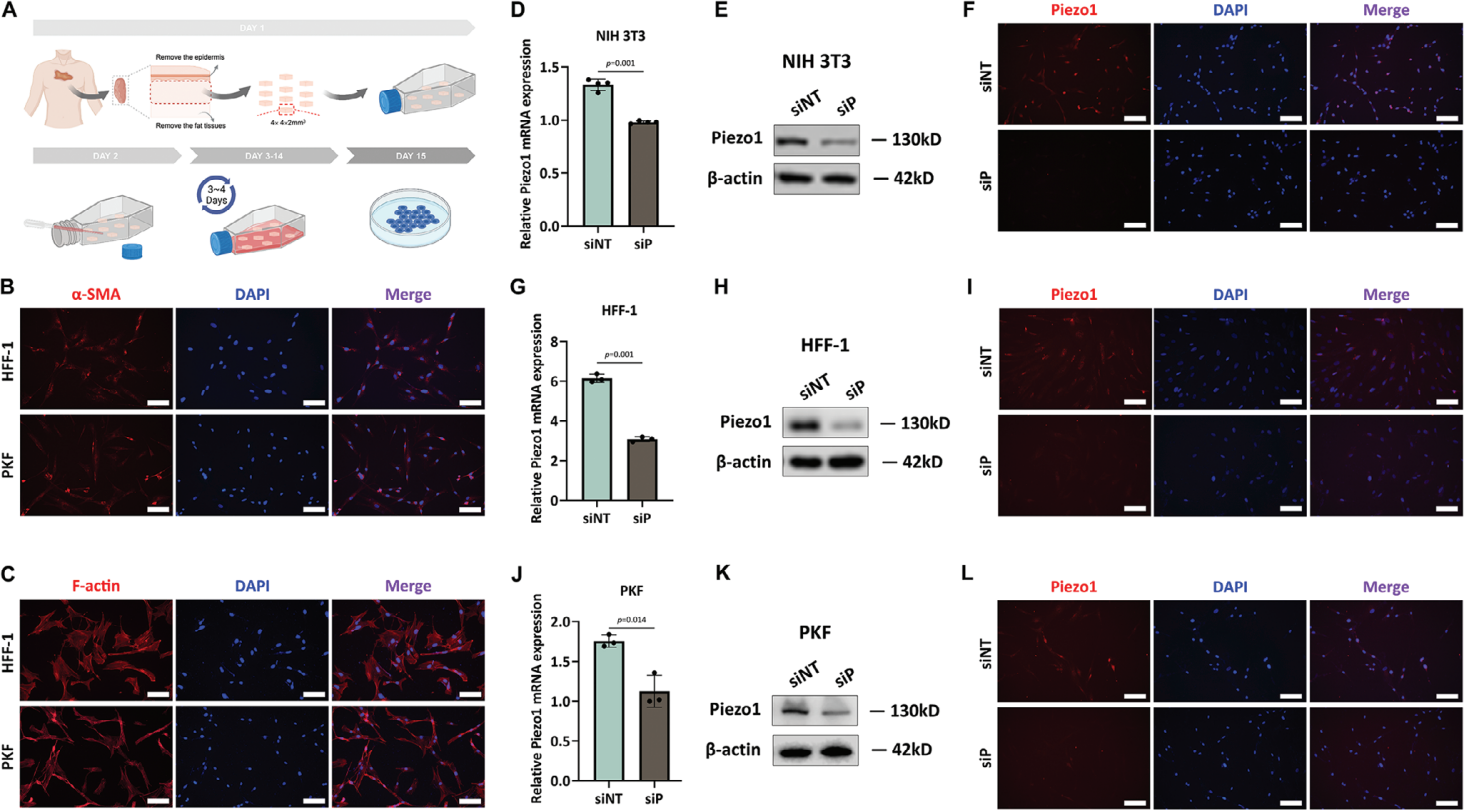
Expression of Piezo1 in fibroblasts. A) Schematic diagram showing the extraction and culture of primary keloid fibroblasts (PKF). B) The expression of α‐SMA (red) in HFF‐1 and PKF. C) The expression of F‐actin (red) in HFF‐1 and PKF. D) Relative Piezo1 mRNA expression in NIH 3T3 and siRNA‐Piezo1 NIH 3T3 cells with and without LFS (n = 4, Student's *t*‐test). E) Western blotting analysis of Piezo1 in NIH 3T3 and siRNA‐Piezo1 NIH 3T3 cells. F) The expression of Piezo1 in NIH 3T3 and siRNA‐Piezo1 NIH 3T3 cells. G) Relative Piezo1 mRNA expression in HFF‐1 and siRNA‐Piezo1 HFF‐1 cells with and without LFS (n = 3, Student's *t*‐test). H) Western blotting analysis of Piezo1 in HFF‐1 and siRNA‐Piezo1 HFF‐1 cells. I) The expression of Piezo1 in HFF‐1 and siRNA‐Piezo1 HFF‐1 cells. J) Relative Piezo1 mRNA expression in PKF and siRNA‐Piezo1 PKF cells with and without LFS (n = 3, Student's *t*‐test). K) Western blotting analysis of Piezo1 in PKF and siRNA‐Piezo1 PKF cells. L) The expression of Piezo1 in PKF and siRNA‐Piezo1 PKF cells. siNT: no target siRNA; siP: siRNA‐Piezo1. Scale bar, 100 µm.

**Figure 4:**
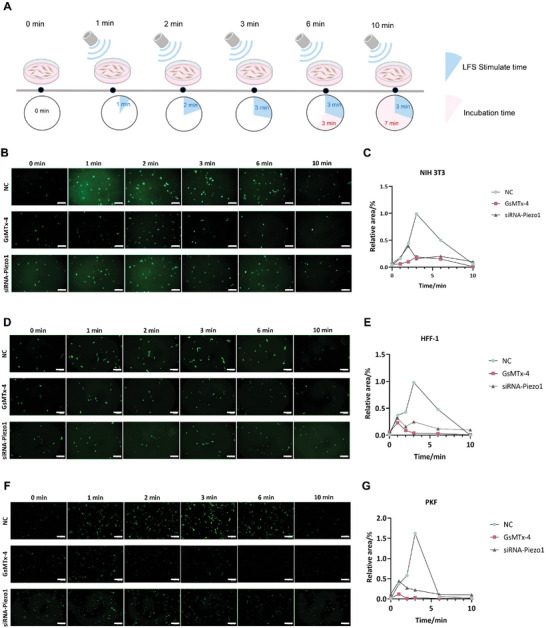
Calcium influx induced by ultrasound via Piezo1 channels in fibroblasts. A) Schematic diagram showing the intervention schedules. Intracellular calcium levels were labeled by Fluo‐8 (green) in B) NIH 3T3, C) HFF‐1, and D) PKF cells after different interventions. Quantitative curve of intracellular calcium levels in E) NIH 3T3, F) HFF‐1, and G) PKF cells after different interventions. Scale Bar, 100 µm.

To gain a deeper insight into keloids, we extracted and cultured patient‐derived primary keloid fibroblasts (PKF). Keloid tissues were obtained from the Department of Dermatology, Xiangya Hospital, Changsha, China (ID: 202 305 092). The protocol for PKF extraction was performed as described by He et al.,^[^ ^28^ ^]^ with several modifications (Figure 4A). To identify the extracted cells type as fibroblasts, immunofluorescence staining of α‐SMA and F‐action were performed (**Figure** **3**B,C). The results showed that PKF had a morphology similar to that of HFF‐1, a proven and widely used human skin fibroblast cell line. Subsequently, we investigated the cellular location of Piezo1. The expression and localization of Piezo1 were identified using qRT‐PCR and immunofluorescence (Figure 3D–I). Piezo1 was expressed in NIH 3T3, HFF‐1, and PKF cells and localized in the plasma membrane and nucleus (Figure 3E,G,I). Three days after Piezo1 siRNA transfection, the mRNA and protein expression levels significantly decreased. We examined the expression of Piezo1 in these three types of fibroblasts and demonstrated that siRNA‐Piezo1 successfully downregulated the expression of Piezo1.

### 2.4 LFS Activates Piezo1‐Regulated Calcium Influx

As a mechanosensitive Ca^2+^ channel, Piezo1 is activated by Ca^2+^ influx. Fluo‐8 AM, a green fluorescent calcium‐binding dye, was used to visualize the LFS‐induced calcium influx. To determine the optimal time point for observation, we first treated PKF with different LFS stimulation and incubation times (Figure S5A, Supporting Information). The prolonging of LFS stimulation time, the Fluo‐8 labeled cells increased with prolonged LFS stimulation (Figure S5B, Supporting Information). Interestingly, calcium influx slowly attenuated when LFS stimulation ceased (Figure S5C, Supporting Information), indicating that part of the Piezo1 channels remained open. However, 4 min after LFS, the green fluorescence signal was barely observed (Figure S5D, Supporting Information). Based on these results, we defined three phases: opening, declining, and closing. Therefore, we selected six time points (t = 0, 1, 2, 3, 6, and 10 min) for subsequent experiments (**Figure** **4**A). Consistent results were observed in NIH 3T3 and HFF‐1 cells (Figure 4B–E). GsMTx‐4 (an inhibitor of Piezo1 channels) and siRNA‐Piezo1 were used to investigate whether LFS‐induced calcium influx was regulated by Piezo1 channels. GsMTx‐4 inhibited 80.8% LFS‐induced calcium influx in NIH 3T3 cells, 76.5% in HFF‐1 cells, and 92.6% in PKF. siRNA‐Piezo1 application suppressed 60.6%, 67.3%, and 72.8% of LFS‐induced calcium influx in NIH 3T3, HFF‐1, and PKF, respectively. The results showed that GsMTx‐4 inhibited LFS‐induced calcium influx more than siRNA‐Piezo1, demonstrating that other mechanosensitive Ca^2+^ channels may play a role in LFS‐induced calcium influx but not as important as Piezo1. Moreover, Piezo1 in PKF was possibly more sensitive to LFS than in normal fibroblasts, as the application of either GsMTx‐4 or siRNA‐Piezo1 suppressed more LFS‐induced calcium influx in PKF than in NIH 3T3 and HFF‐1 cells.

### 2.5 LFS Inhibits the Migration of Fibroblasts in a Piezo1‐Regulated Manner

**Figure 5:**
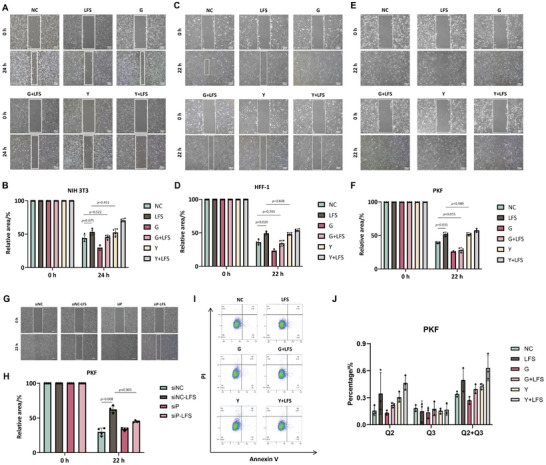
Inhibited migration of fibroblasts and increased apoptosis of PKF by LFS via Piezo1 channels. A) Randomly selected images of the gap of NIH 3T3 cells at 0 and 24 h after LFS. B) Relative area of the gap in NIH 3T3 over time. Randomly selected images of the gap of NIH 3T3 cells at 0 and 24 h after LFS (n = 4, Student's *t*‐test). C) Randomly selected images of the gap of HFF‐1 cells at 0 and 22 h after LFS. D) Relative area of the gap in HFF‐1 over time (n = 3, Student's *t*‐test) E, G) Randomly selected images of the gap of PKF cells at 0 and 22 h after LFS. Scale bar: 200 µm. F, H) Relative area of the gap in PKF over time (F: n = 3, H: n = 4, Student's *t*‐test). I) Apoptosis detection of PKF cells by flow cytometry. J) Quantitative analysis of early apoptosis, advanced apoptosis, and total apoptosis of PKF cells (n = 3, Student's *t*‐test). G: GsMTx‐4, Y: Yoda1, siNC: siRNA‐NC, siP: siRNA‐Piezo1, Scale bar, 200 µm.

To explore whether Piezo1 participates in LFS‐induced inhibition of migration, we performed a scratch wound healing assay on fibroblasts after different interventions. Images of the scratch wound and quantitative visualization are shown in **Figure** **5**. We observed delayed healing of scratch wounds in LFS‐treated NIH 3T3 (Figure 5A,B, *p* = 0.075), HFF‐1 (Figure 5C,D, *p* = 0.020), and PKF cells (Figure 5E,F, *p* = 0.031). We used the inhibitor GsMTx‐4 (G) and the activator Yoda1 (Y) of Piezo1 to investigate its role Piezo1 in this process. Similar results were observed between the NC and G+LFS groups (NIH 3T3: *p* = 0.522; HFF‐1: *p* = 0.705; PKF: *p* = 0.055), indicating that the application of GsMTx‐4 could prevent the LFS‐induced inhibition of migration. The performances of the LFS and Y groups were semblable (NIH 3T3: *p* = 0.431; HFF‐1, *p* = 0.608; PKF, *p* = 0.989). As shown in Figure 5G,H, the effect of LFS on PKF migration was significantly reduced by the knockdown of the Piezo1 channel (but was not significant in NIH 3T3 or HFF‐1 cells). These results suggested that the inhibition of migration by LFS may be regulated by Piezo1 channels in fibroblasts. Notably, the migration ability of PFK cells in the G+LFS group was as strong as that in the G group (Figure 5E,F, *p* = 0.437), which was not observed in NIH 3T3 (Figure 5A,B, *p* = 0.014) or HFF‐1 cells (Figure 5C,D, *p* = 0.011). This indicates that Piezo1 in PKF cells may play a more important role in LFS‐induced inhibition of migration than in normal fibroblast cell lines.

### 2.6 LFS Improves the Apoptosis of PKF in a Piezo1‐Regulated Manner

Next, we examined the role of Piezo1 in LFS‐induced apoptosis. Flow cytometry was used to analyze apoptosis. Before LFS stimulation, we treated NIH 3T3, HFF‐1, and PKF with GsMTx‐4 or Yoda1. As expected, early apoptosis and total apoptosis rates both increased after LFS in PKF (Figure 5I,J). GsMTx‐4 treatment restrained LFS‐induced apoptosis, while Yoda1 increased the early and total apoptosis rates of PKF. These results showed that Piezo1 activation may regulate LFS‐induced PKF apoptosis. However, LFS only slightly increased early and total apoptosis of HFF‐1 cells (Figure S6, Supporting Information). Moreover, the apoptosis of NIH 3T3 cells was inhibited by LFS (Figure S7, Supporting Information). Though GsMTx‐4 and Yoda1 influenced the early and total apoptosis of NIH 3T3 and HFF‐1 cells, the results were not consistent with those of PKF. Similar to the results in Sections 2.4 and 2.5, one of the possible reasons was that Piezo1 in PKF was more sensitive to LFS stimulation than in NIH 3T3 and HFF‐1 cells. Collectively, these results indicated that LFS increased the early and total apoptosis of PKF, which was regulated by Piezo1 channels. In addition to migration and apoptosis, we observed in 2.2 that LFS inhibited collagen synthesis in NIH 3T3 cells. However, the role of Piezo1 in this process remains unclear as neither GsMTx‐4 nor Yoda1 influenced the results.

### 2.7 In Vivo Efficacy of LFS in Treating Keloids Regulated by Piezo1

**Figure 6:**
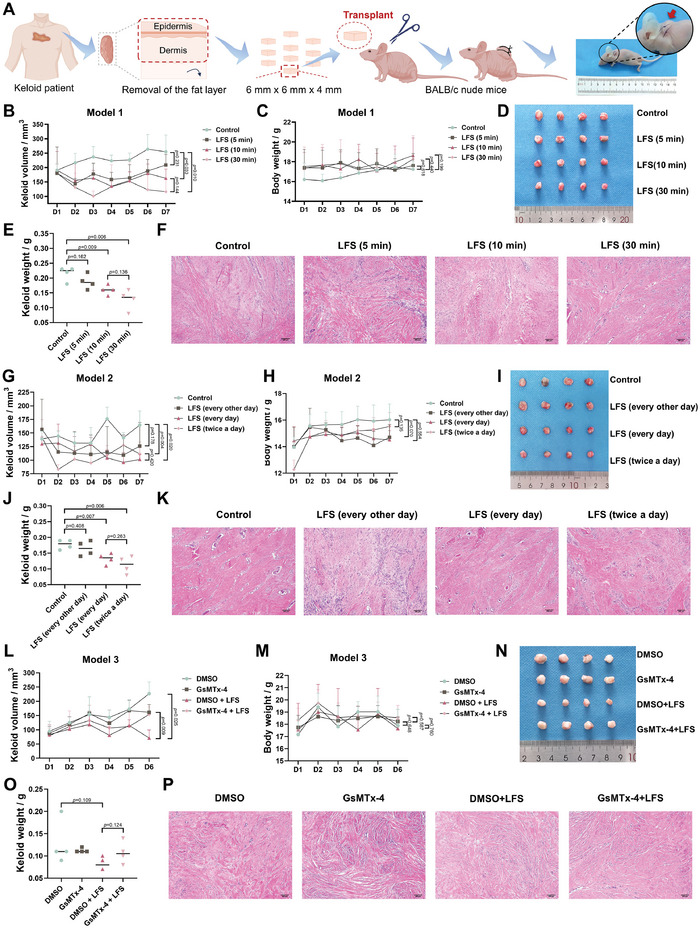
The efficacy of LFS in keloid‐bearing mice model. A) The flow chart of establishing patient‐derived xenograft keloid‐implantation mice model. The volume of keloids in B) Model 1, G) Model 2, and L) Model 3. The body weight of mice in C) Model 1, H) Model 2, and M) Model 3. The images of keloids in D) Model 1, I) Model 2, and N) Model 3. The weight of keloids in E) Model 1, J) Model 2, and O) Model 3. The H&E staining images of F) Model 1, K) Model 2, and P) Model 3. Scale bar: 200 µm.

To further verify the in vivo efficacy of LFS for keloid treatment, we established a patient‐derived xenograft keloid implantation mouse model (**Figure** **6**A). Considering the potential heterogeneity between in vitro and in vivo keloid models, the optimal LFS treatment time and frequency were investigated in two keloid‐bearing mouse models. In Model 1, we designed four groups with different LFS treatment times: Group 1, control; 0 min; Group 2,5 min; Group 3,10 min; and Group 4,30 min. LFS was administered to the mice every day for 6 days. On day 7, the mice were euthanized. Compared with the control group, the efficacy of the 5‐min LFS treatment in treating keloids was not significant (volume: *p* = 0.231, weight: *p* = 0.162), although the volume and weight of keloids in Group 2 were slightly decreased (Figure 6B–D). The volume and weight of keloids significantly decreased in the 10‐min (volume: *p* = 0.033, weight: *p* = 0.009) and 30‐min LFS (volume: *p* = 0.010, weight: *p* = 0.006) treatment groups. However, there was no significant difference between the 10‐min and 30‐min treatment groups (volume, *p* = 0.144; weight, *p* = 0.136).

Based on the results of Model 1, we selected 10 min as the optimal single treatment time for keloid‐bearing mice. To further investigate the optimal treatment frequency, Model 2 was established, and four groups were designed: Group 1, control; no treatment; Group 2,10‐min LFS undertaken every other day; Group 3:10‐min LFS undertaken every day; Group 4:10‐min LFS undertaken twice a day (with an interval of 4 h). Compared with no treatment, the 10‐min LFS treatment showed no significant improvement in keloid volume (*p* = 0.178) or weight (*p* = 0.408). However, daily and twice‐daily LFS treatments significantly decreased the volume (*p* = 0.004 and *p* = 0.020, respectively) and weight (*p* = 0.007 and *p* = 0.006, respectively) of the keloids. Notably, the efficacy of twice‐daily LFS treatment in keloid‐bearing mice did not significantly exceed LFS treatment once daily. In Models 1 and 2, the body weights of the keloid‐bearing mice in each group were comparable. Hematoxylin and eosin staining demonstrated that the organization of the keloid dermis became loose with increased LFS time and frequency.

The results of Models 1 and 2 demonstrated the effectiveness of LFS in treating keloids in vivo. To verify whether Piezo1 channels regulate the in vivo efficacy of LFS, GsMTx‐4 (5 µm, 20 µL) was injected intralesionally in keloids in Model 3 to block Piezo1 channels. Following a 30‐min incubation with GsMTx‐4, a 10‐min LFS treatment was performed. Compared to the intralesional injection of DMSO (working concentration: <5%), the solvent of GsMTx‐4, the efficacy of LFS in decreasing keloid volume was significantly impaired by the application of GsMTx‐4 (*p* = 0.009). The body weights of the keloid‐bearing mice were not significantly different among the groups.

## 3 Discussion

**Figure 7:**
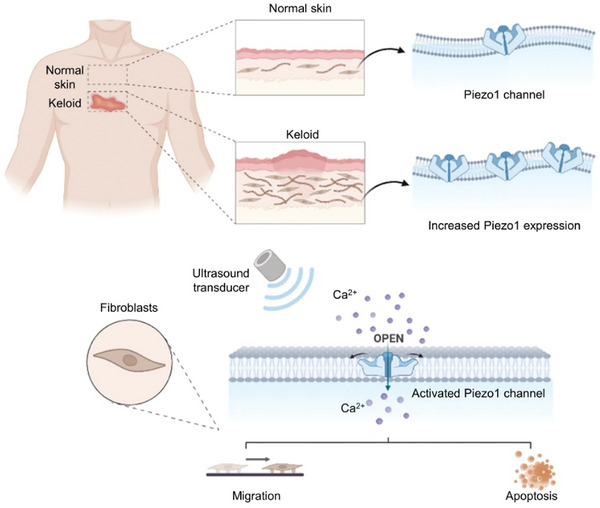
The schematic diagram of increased Piezo1 expression in keloid and enhanced calcium influx by LFS stimulation in a Piezo1‐regulated manner.

Our results showed that Piezo1 expression increased in keloids than in healthy skin or normal scars. Piezo1 channels are present in the cytomembrane and perinuclear space of fibroblasts. To gain deeper insight into keloids, we extracted patient‐derived PKF. It has been observed that LFS activates Piezo1 and enhances Piezo1‐induced calcium influx. Furthermore, these effects persist even after the cessation of LFS, which can be termed the post‐LFS effect. The migration of both normal skin fibroblasts and PKF was inhibited, and the apoptosis of PKF was promoted by LFS treatment in a Piezo1‐regulated manner (**Figure** **7**). The efficacy of LFS in decreasing the volume and weight of keloids was consistently demonstrated in a human keloid‐bearing mouse model. The application of GsMTx‐4, a selective inhibitor of the Piezo1 channel, impaired the in vivo effects of LFS. To the best of our knowledge, this is the first study to reveal the influence of low‐frequency ultrasound on keloids and the role of Piezo1 in this process.

Studies using ultrasound in keloids have primarily focused on the assessment of severity and drug delivery for anti‐keloid therapy.^[^ ^29^, ^30^ ^]^ However, the efficacy of ultrasound as a standalone treatment for keloids remains undetermined, and the mechanism of ultrasound‐induced stimulation in fibroblasts requires further investigation. Here, the migration inhibition, collagen synthesis suppression, and apoptosis‐promoting effects of LFS monotherapy in fibroblasts were determined. To further investigate the effects of LFS on keloids, we performed an animal experiment using a patient‐derived xenograft keloid implantation mouse model. Compared with no treatment, 10‐min of LFS treatment for six consecutive days significantly decreased the volume and weight of keloids, indicating that LFS can inhibit the malignant function of keloids at the laboratory level.

Piezo1 is a mechanosensitive and nonselective cation channel that responds to mechanical stimuli by deforming from a bowl‐shaped trimer toward a planar structure.^[^ ^31^ ^]^ Several studies have demonstrated that Piezo1 is an important mediator of the ultrasonic modulatory effect, which can be activated by ultrasound to trigger downstream signaling pathways (Table S1, Supporting Information). Qiu et al. discovered that ultrasound upregulates the expression of several proteins involved in complex neuronal functions in a Piezo1‐regulated pathway.^[^ ^23^ ^]^ Zhang G et al. proposed that low‐intensity pulsed ultrasound promotes the migration of osteoblastic cells by activating Piezo1.^[^ ^21^ ^]^ Singh et al. showed that ultrasound could enhance tumor cell death by activating Piezo1 channels.^[^ ^32^ ^]^ Our study demonstrated that LFS inhibits fibroblast migration and increases PKF apoptosis by activating Piezo1 channels in vitro. Topical application of GsMTx‐4 in keloid‐bearing mice can impair the efficacy of LFS in vivo. The activation of Piezo1 has been proven to have implications for scar treatment. It has been demonstrated that agonist‐induced Piezo1 activation suppresses the migration of transformed fibroblasts.^[^ ^33^ ^]^ Moreover, Zhang et al. discovered that ultrasound can downregulate the expression of Piezo1, and ultrasound‐induced Piezo1 downregulation promotes neurogenesis and inhibits inflammation.^[^ ^34^ ^]^ In our study, we did not observe any significant changes in the expression level of Piezo1 following LFS.

Additionally, we compared Piezo1 expression in keloid and normal scars based on publicly available single‐cell RNA‐seq data. These results suggested that Piezo1 is upregulated in keloids. Previous studies have concluded that regions exhibiting higher Piezo1 expression demonstrate an increased response to ultrasound.^[^ ^35^ ^]^ We also observed the targeted effects of LFS on PKF. The LFS‐induced inhibition of migration induced by LFS is more significant in PKF cells than in NIH 3T3 or HFF‐1 cells. The apoptosis‐inducing effect of LFS was only observed in PKF. The LFS‐based apoptosis of PKF is consistent with several studies conducted on tumors, which have reported that abnormal cells are more sensitive to mechanical stress.^[^ ^36^, ^37^ ^]^ The current understanding of how mechanical stress leads to an elevation in cytoplasmic calcium levels, subsequently triggering tumor cell apoptosis, remains insufficient.^[^ ^38^ ^]^ In this study, we propose that one possible reason is that the expression of Piezo1 is higher in PKF, which enables them to be more sensitive to ultrasound stimuli. As an important secondary messenger, the formation of higher intracellular Ca^2+^ concentrations in PKF may trigger a series of downstream cascade amplification reactions, such as the Piezo1‐Ca^2+^‐MLCK and RAF‐MEK‐ERK signaling pathways.^[^ ^39^, ^40^ ^]^ Accumulated alterations of these pathways mediate significant differences in biological behavior between PKF and normal fibroblasts. Therefore, it is rational to consider utilizing the high expression of Piezo1 in keloids and low‐frequency ultrasound to provide a noninvasive strategy for treating keloids from the perspective of mechanotransduction. This study has some limitations that should be acknowledged. First, the signaling pathway between Piezo1‐induced Ca^2+^ influx and functional alterations in fibroblasts remains incompletely explored. Second, additional research involving in vivo experiments with larger sample sizes is necessary to better translate these findings to clinical applications.

## 4 Conclusion

Our study investigated the potential application of LFS in controlling patient‐derived fibroblast migration and apoptosis in vitro and inhibiting keloid growth in vivo. We propose that Piezo1 channels are activated by LFS and are involved in the LFS‐induced inhibition of cell migration and validity. Furthermore, ultrasound should not be viewed solely as a skin permeation device but rather as an intervention capable of independently modulating the functionality of keloids. It has potential as a therapeutic approach that can directly affect keloid behavior, leading to improved treatment outcomes for keloid management.

## 5 Experimental Section

### Fabrication Of Ultrasound Transducer

A non‐conductive resin (elastic modulus of 3.8 GPa, hardness of 86 shore D) was chosen for use in the front end, and Aluminum for the front linking. The two parts were made by 3D‐printed technology. The PZT sheets utilized the piezoelectric material (PZT8) which exhibits excellent piezoelectric properties, high electromechanical coupling coefficient, and low dielectric loss. The backing was made of 45# steel. The PZT sheets and copper electrodes were clamped to the front end and the backing with the pre‐tightening nuts.

### Finite Element Analysis

Finite element model of acoustic field was established using the commercial finite element software COMSOL Multiphysics 6.0. The water area was a 35 mm diameter hemisphere and the thickness of the perfect match layer (PML) was 20 mm. The mesh model has 66 128 elements and 11 070 nodes. Boundary conditions were chosen to match the experimental conditions. To achieve amplitude superposition, one of the PZT disks was poled along the +Z direction while another was poled along the −Z direction. A 200 Vpp sinusoidal signal was applied to the PZT disks.

### Cell Lines and Cell Culture

NIH 3T3 and HFF‐1 cell lines and PKF were cultured in DMEM (BasalMedia, L110KJ) supplemented with 10% FBS (CellMax, SA101) and 1% penicillin‐streptomycin solution (BI, 03‐031‐1B). Cells were cultured in an incubator at 37 °C and under 5% CO_2_. Cells were subcultured via trypsinization once reaching confluency of ≈90%. Passage 3–6 cells were used for experiment.

### Isolation of Primary Keloid Fibroblast (PKF)

After obtaining ethical approval (ID: 202305092), human keloid tissues were obtained from the Department of Dermatology, Xiangya Hospital, Central South University. The human keloid tissues were soaked in Dulbecco's Modified Eagle Medium (DMEM) (BasalMedia, L110KJ) and transported to the laboratory at 4 °C. In an ultra‐clean workbench, the epidermis and fat layer of keloid tissues were removed. The remained dermis was cut into 4 mm × 4 mm × 2 mm pieces and placed in 25 cm culture flasks (Excell, CS016‐0104). DMEM (BasalMedia, L110KJ) (50 µL) supplemented with 10% FBS (CellMax, SA101) and 1% penicillin‐streptomycin solution (BI, 03‐031‐1B) was added to each piece. Tissues were cultured in an incubator at 37 °C and under 5% CO_2_. The next day, 2–3 mL complete medium was added to each culture flask. The medium was changed every 3 days. After 15 days, most of PKF had crawled out of the tissues and were subcultured via trypsinization.

### Scratch Wound Healing Assay

NIH 3T3, HFF‐1, PKF cells were seeded in 6‐well plates at 4 × 10^5^ cells per well. After 24 h, the scratch wound was made by 10 µL tips. Cell debris was softly flushed off by DPBS. The plating medium was removed and replaced with DMEM supplemented with 2% FBS. Images were collected by microscope (Nikon). at different time points. The collection of images was discontinued once the scratch wounds in the negative control group had completely healed.

### Data Collection and scRNA‐Seq Analysis

Single‐cell RNA‐seq data of keloid and normal scar dermis tissues was from a previously published article conducted by Deng C et al.^[^ ^19^ ^]^ (GSE163973, 40655 single cells isolated from 3 kelnoid sample vs 3 normal scar dermis tissues). The R package “Seurat”^[^ ^41^ ^]^ (Version 4.0.4) was used to perform single‐cell transcriptomic analysis. Uniform manifold approximation and projection (UMAP) was used in scRNA‐seq for dimension reduction and visualization.^[^ ^42^ ^]^ Two groups of fibroblasts with high expression and non‐expression of PIEZO1 were distinguished based on UMI‐count to determine a specific gene expression. Enrichment score of Reactome pathway was calculated by using gene set variation analysis (GSVA)^[^ ^43^ ^]^ and cluster Profiler (Version 4.0.4).^[^ ^44^ ^]^

### Immunohistochemistry Staining and Imaging

Keloids and normal skin tissues were collected from the Department of Dermatology, Central South University after ethical approval was obtained (ID: 202305092). NIH 3T3, siRNA‐Piezo1 NIH 3T3, HFF‐1, siRNA‐Piezo1 HFF‐1, PKF, and siRNA‐Piezo1 PKF were seeded into 6‐well plates at 1–2 × 10^5^ cells per well and maintained in an incubator at 37 °C and 5% CO_2_ overnight. The next day, the cells were rinsed with Dulbecco's phosphate‐buffered saline (DPBS) (BI, 02‐023‐1A) twice and fixed with 4% paraformaldehyde (Servicebio, G1101‐500ML) for 20 min at room temperature. After this, the cells were permeabilized by 0.5% Triton X‐100 (Sangon Biotech, A600198‐0500) for 10 min and were blocked by 5% bovine serum albumin (BSA) (Genview, FA016) in PBS for 2 h at room temperature. Subsequently, the cells were incubated with a primary antibody against α‐SMA (1:1000, protein tech, 14395‐1‐AP) and Piezo1 (1:200, LSBio, LS‑C153413) at 4 °C overnight. After washing three times with PBS, the cells were protected from light and incubated with a secondary antibody (Alexa Fluro 594 donkey Anti‐rabbit) (1:1000, Invitrogen, A21207) for 1 h at room temperature. The cells were washed five times with PBS and then incubated with fluoro shield mounting medium with DAPI (Abcam, ab104139) for 10 min at room temperature. Following staining, the cells were imaged using a fluorescence microscope (Nikon, ECLIPSE Ts2R).

### Quantitative Real‐Time PCR (qRT‐PCR)

Total RNA was extracted by Magzol reagent (Magen, R4801‐01) according to the manufacturer's protocol. cDNA was synthesized using a Hifair III 1st Strand cDNA Synthesis SuperMix for qPCR Kit (Yeasen, 11141ES60). Quantitative PCR was performed using 2X SYBR Green qPCR Master Mix (Bimake, B21703) following the manufacturer's guidelines. GAPDH was used as the internal control. Primers were bought from Sangon Biotech and the sequences of primers were as follows. Mouse GAPDH: GCACCACCAACTGCTTAG (forward, 5′−3′), GGATGCAGGGATGATGTTC (reverse, 5′−3′); mouse Piezo1: CTTACACGGTTGCTGGTTGG (forward, 5′−3′), CACTTGATGAGGGCGGAAT (reverse, 5′−3′); human GAPDH: AGCCACATCGCTCAGACAC (forward, 5′−3′), GCCCAATACGACCAAATCC (reverse, 5′−3′); human Piezo1: CCACCAACCTCATCAGCGACTT (forward, 5′−3′), ACCAGCACCAGCCAGAACAG (reverse, 5′−3′).

### F‐Actin Staining

HFF‐1 and PKF were seeded into 6‐well plates at 1–2×10^5^ cells per well and maintained in an incubator at 37°C and 5% CO_2_ overnight. After being rinsed by DPBS twice, cells were fixed by 4% paraformaldehyde for 20 min and permeabilized using 0.5% Triton X‐100 for 10 min at room temperature. Then, cells were protected from light and incubated with a mix of 1µL 1000 × Rhodamine Phalloidin (MedChemExpress, HY‐K0903) and 1 mL PBS containing 1%BSA for 30 min at room temperature. After being washed with PBS three times, the cells were incubated with DAPI for 10 min at room temperature. Following staining, the cells were imaged using a fluorescence microscope (Nikon, ECLIPSE Ts2R).

### Immunofluorescence Staining and Imaging

NIH 3T3, siRNA‐Piezo1 NIH 3T3, HFF‐1, siRNA‐Piezo1 HFF‐1, PKF, and siRNA‐Piezo1 PKF were seeded into 6‐well plates at 1–2 × 10^5^ cells per well and maintained in an incubator at 37 °C and 5% CO_2_ overnight. The next day, the cells were rinsed with Dulbecco's phosphate‐buffered saline (DPBS) (BI, 02‐023‐1A) twice and fixed with 4% paraformaldehyde (Servicebio, G1101‐500ML) for 20 min at room temperature. After this, the cells were permeabilized by 0.5% Triton X‐100 (Sangon Biotech, A600198‐0500) for 10 min and were blocked by 5% bovine serum albumin (BSA) (Genview, FA016) in PBS for 2 h at room temperature. Subsequently, the cells were incubated with a primary antibody against α‐SMA (1:1000, protein tech, 14395‐1‐AP) and Piezo1 (1:200, LSBio, LS‑C153413) at 4 °C overnight. After washing three times with PBS, the cells were protected from light and incubated with a secondary antibody (Alexa Fluro 594 donkey Anti‐rabbit) (1:1000, Invitrogen, A21207) for 1 h at room temperature. The cells were washed five times with PBS and then incubated with fluoro shield mounting medium with DAPI (Abcam, ab104139) for 10 min at room temperature. Following staining, the cells were imaged using a fluorescence microscope (Nikon, ECLIPSE Ts2R).

### Piezo1 siRNA Transfection

NIH 3T3, HFF‐1, and PKF were plated into 6‐well plates at 1.5 × 10^5^ cells per well 24 h prior to siRNA transfection. After the cells reached ≈40% confluence, the complete medium was replaced with a mix of 2 mL DMEM, 6 µL lipofectamine 2000 transfection reagent (Therma Fisher, 11668019), and 12 µL (5OD mL^−1^) siRNA (GenePharma). For NIH 3T3: no target siRNA: UUCUCCGAACGUGUCACGUTT (sense, 5′−3′), ACGUGACACGUUCGGAGAATT (antisense, 5′−3′); siRNA‐mouse Piezo1: CCGGCAUCUACGUCAAAUATT(sense1, 5′−3′), UAUUUGACGUAGAUGCCGGUG (antisense1, 5′−3′); CAAGAAAUACAACCAUCUATT (sense2, 5′−3′), UAGAUGGUUGUAUUUCUUGGU (antisense2, 5′−3′);

GGCGCUUGCUAGAACUUCATT (sense3, 5′−3′), UGAAGUUCUAGCAAGCGCCGA (antisense3, 5′−3′). For HFF‐1 and PKF: no target siRNA: UUCUCCGAACGUGUCACGUTT (sense, 5′−3′), ACGUGACACGUUCGGAGAATT (antisense, 5′−3′); siRNA‐human Piezo1: UGGAGUAUGCCAACGAGAATT (sense1, 5′−3′), UUCUCGUUGGCAUACUCCATT (antisense1, 5′−3′); GCAGCAUGACAGACGACAUTT (sense2, 5′−3′), AUGUCGUCUGUCAUGCUGCTT (antisense2, 5′−3′); GCGCAUCAGUCUACGUUUUTT (sense3, 5′−3′), AAAACGUAGACUGAUGCGCTT (antisense3, 5′−3′). After incubation for 4–6 h, the mix was replaced by a complete medium and the cells were cultured in an incubator at 37 °C and 5% CO_2_.

### Inhibition and Activation of Piezo1 Channel

Piezo1‐induced cation influx was inhibited by the incubation of 500 nm GsMTx‐4 (Abcam, ab141871) for 30 min at 37 °C and 5% CO_2_. The activation of Piezo1 was induced by 5 µm Yoda1 (MedChemExpress, 448947‐81‐7) for 5 min at 37 °C and 5% CO_2_.

### Fluorescence Imaging of Cellular Ca^2+^

Cells were plated in 3.5 cm culture dishes (Excell, CS016‐0124) at 3 × 10^5^ cells per dish and maintained in an incubator at 37 °C and 5% CO_2_ overnight. After 24 h, cells were incubated with DPBS containing 1 µg mL^−1^ Fluo‐8 AM (Abcam, AB142773) for 1 h with or without 30‐min incubation of 500 nm GsMTx‐4 (Abcam, ab141871) at 37 °C. Before intervention, the mix was removed and rinsed with DPBS three times. Then, a complete medium was added to the dish to provide extracellular calcium. After ultrasound stimulation, cells were quickly washed by DPBS once and fixed with 4% paraformaldehyde for 20 min at room temperature.

### Apoptosis Detection

Following LFS intervention, cells were maintained in an incubator at 37 °C and 5% CO_2_ for 24 h before apoptosis detection. Cells were digested by trypsin solution without EDTA (Beyotime, C0205) and rinsed by cold DPBS. Annexin V Alexa Fluor647/PI Apoptosis Detection Kit (4A Biotech, FXP023‐100) was applied according to the manufacturer's protocol.

### Animal Model

Female New Zealand rabbits aged 4 weeks (weight: 2.0–2.5 kg) were purchased from the Hunan Slac Laboratory Animal (Slac, Hunan, China). All procedures were approved by the Animal Care and Use Committee of Central South University (CSU‐2022‐0555). A hypertrophic scar model was established on the ventral side of the rabbit ear according to the previously reported protocol with modifications.^[^ ^45^ ^]^ After routine anesthesia and disinfection, five full‐thickness skin resections (1 cm × 1 cm) were created on each ear, and the perichondrium on the base of the wound was completely removed at day 0. Seven days after the surgery, the slabs were ripped off. Twenty‐one days after the surgery, a raised hypertrophic scar replaced the wound.

### Western Blot

Cells were lysed in RIPI Lysis Buffer (Beyotime) supplemented with protease inhibitors and phosphatase inhibitors (Bimake). Protein concentrations were measured with the BCA reagent (Beyotime) by using a Beckman Coulter DU‐800 spectrophotometer. Equal amounts of protein were resolved by SDS‐PAGE and immunoblotted with Piezo1 Monoclonal Antibody (Invitrogen, MA5‐32876) at a dilution of 1:1000. The immunoblots were detected using an Odyssey Fc Imaging System (LI‐COR).

### Patient‐Derived Xenograft Keloid‐Implantation Mouse Model

The protocol of establishing a patient‐derived xenograft keloid‐implantation mouse model referred to previously published research and was modified.^[^ ^46^ ^]^ Sixty BALB/c female nude mice at the age of 3–4 weeks were purchased from Hunan SJA Laboratory Animal Co., Ltd and bred in specific pathogen‐free conditions. Specimens from three keloid patients were collected from the Department of Dermatology and the Department of Plastic Surgery, Xiangya Hospital, Central South University with ethical approval (CSU‐2022‐0555). Keloid tissues were placed into 50 mL centrifuge tubes, each containing 20 mL of serum‐free DMEM. The samples were immediately transported to the animal laboratory center at a temperature of 4 °C. After removal of adipose and/or necrotic tissues, the keloid tissues were rinsed in DPBS for two times. Next, the specimens were cut into uniform pieces (6 × 6 × 4 mm^3^) with sterile scissors and scalpel blades. Keloid pieces were then implanted into the upper back of nude mice subcutaneously and the incisions were stitched by 6‐0 suture. Seven days after implantation, the keloid‐bearing mice were ready for LFS treatment.

### I Treatment

Before each LFS treatment, the longest (*L*) and shortest (*S*) diameter of the implantations and body weights of keloid‐bearing mice were measured every day. And the volumes of the xenografts were calculated using the formula *V* = 0.5 × (*L* × *S* ^2^). Two models were established to confirm the in vivo treatment efficacy of LFS and investigate the optimal parameters of LFS. Model 1 was designed to explore the best time (5, 10, and 30 min) duration of LFS treatment, and Model 2 was designed to explore the best treatment frequency (every 2 days, every day, twice a day). The mice were randomly divided into the experimental and the control groups with equal mean volumes of xenografts and body weights of nude mice. For ultrasound treatment, the LFS probe was in contact with the keloid but not compressed, and ultrasound gel was applied between the probe and the mouse keloid to ensure ultrasound coupling. The mice were anesthetized by isoflurane gas before LFS treatment. The keloid‐bearing mice were sacrificed and the keloids were removed on day 7.

### Hematoxylin‐Eosin (H&E) Staining

Tissue sections were dewaxed with environment‐friendly dewaxing reagent (ZSGB‐BIO) and rehydrated with ethanol at different concentrations, and then they were stained with hematoxylin (Thermo) for 3 min and rinsed with running water. After that, they were stained with eosin (Thermo) for 10 s.

### Statistical Analysis

All data were obtained from at least three independent experiments and were expressed as mean ± SD. The exact sample size for each experimental group is shown in every Figure as the number of dots. Two‐tailed unpaired Student's *t*‐test was used to compare the differences between two different treatment groups, and one‐way ANOVA with post hoc tests was used to compare the differences among the three or more different treatment groups. All statistical analyses were accomplished using GraphPad Prism software (v 9.5.1). *p* < 0.05 was considered statistically significant and the exact *p*‐value of each analysis was reported in the corresponding figure.

## Conflict of Interest

The authors declare no conflict of interest.

## Supporting information

Supporting Information

## Data Availability

The data that support the findings of this study are available from the corresponding author upon reasonable request.
