# Supplementary material for: Low‐Frequency Ultrasound Sensitive Piezo1 Channels Regulate Keloid‐Related Characteristics of Fibroblasts
Source: Adv Sci (Weinh). 2024 Feb 4;11(14):2305489. doi: 10.1002/advs.202305489 (PMC11005750; doi:10.1002/advs.202305489)
Supplement: Supplementary file 1 — Supporting Information [file ADVS-11-2305489-s001.pdf]

ADVANCED SCIENCE
Open Access

Supporting Information

for *Adv. Sci.*, DOI 10.1002/advs.202305489

Low-Frequency Ultrasound Sensitive Piezo1 Channels Regulate Keloid-Related Characteristics of Fibroblasts

Zixi Jiang, Ziyan Chen, Yantao Xu, Hui Li, Yixin Li, Lanyuan Peng, Han Shan, Xin Liu, Huayi Wu, Lisha Wu, Dan Jian, Juan Su, Xiang Chen, Zeyu Chen and Shuang Zhao**

ADVANCED SCIENCE
Open Access

Supporting Information

Low-Frequency Ultrasound Sensitive Piezo1 Channels Regulate Keloid-related Characteristics of Fibroblasts

Zixi Jiang, Ziyan Chen, Yantao Xu, Hui Li1, Yixin Li1, Lanyuan Peng, Han Shan Xin Liu1, Huayi Wu, Lisha Wu1, Dan Jian, Juan Su, Xiang Chen, Zeyu Chen*, and Shuang Zhao**

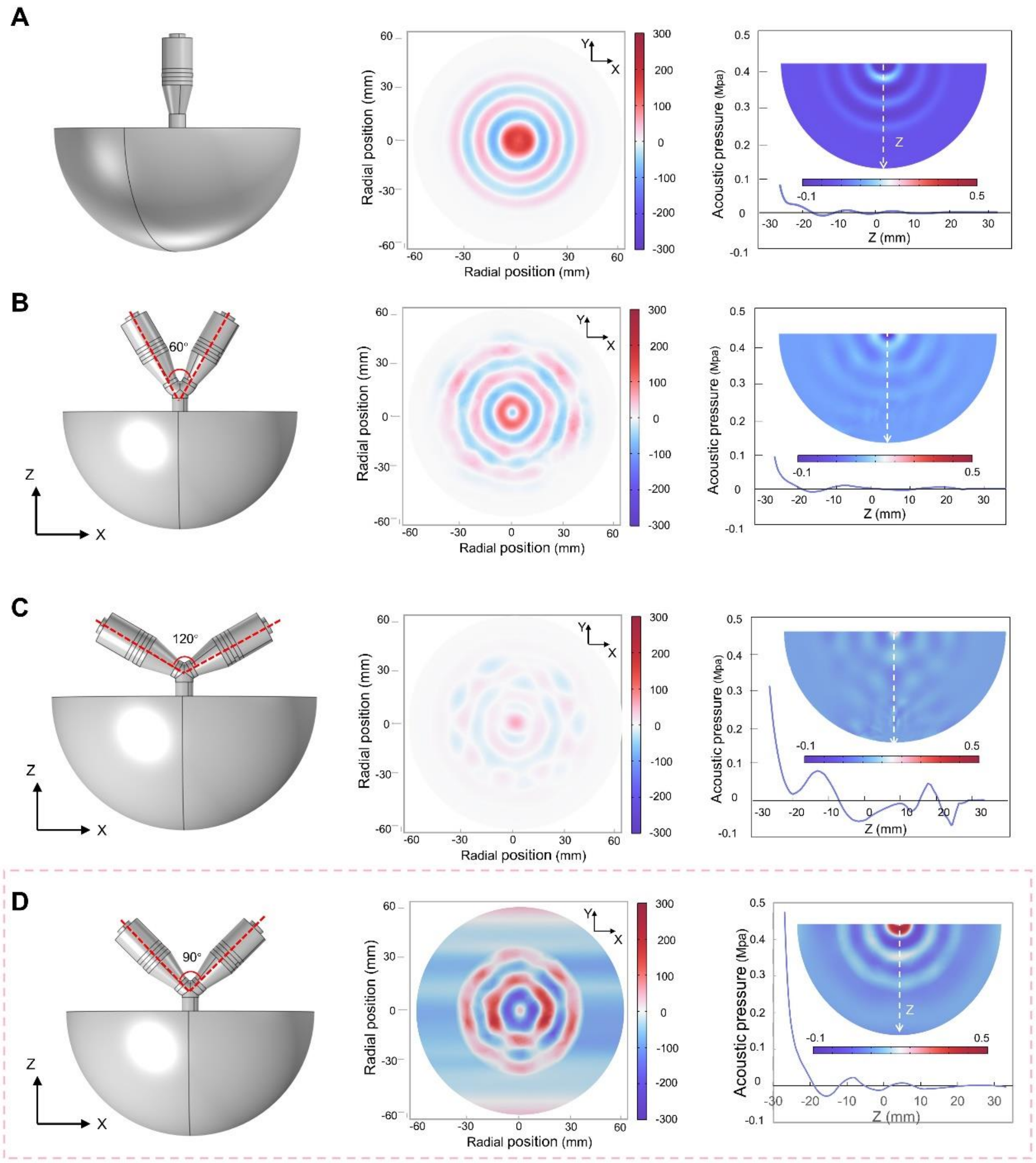

Figure S1. Finite element method (FEM) simulation of the acoustic field under different ultrasound transducer treatments. A) FEM simulation of single probe acoustic pressure contact with the water surface and along the depth direction Z. B) FEM simulation of two probes with an angle of 60° degrees acoustic pressure contact with the water surface and along the depth direction Z. C) FEM simulation of two probes with an angle of 120° degrees acoustic pressure contact with the water surface and along the depth direction Z. D) FEM simulation of two probes with an angle of 90° degrees acoustic pressure contact with the water surface and along the depth direction Z.

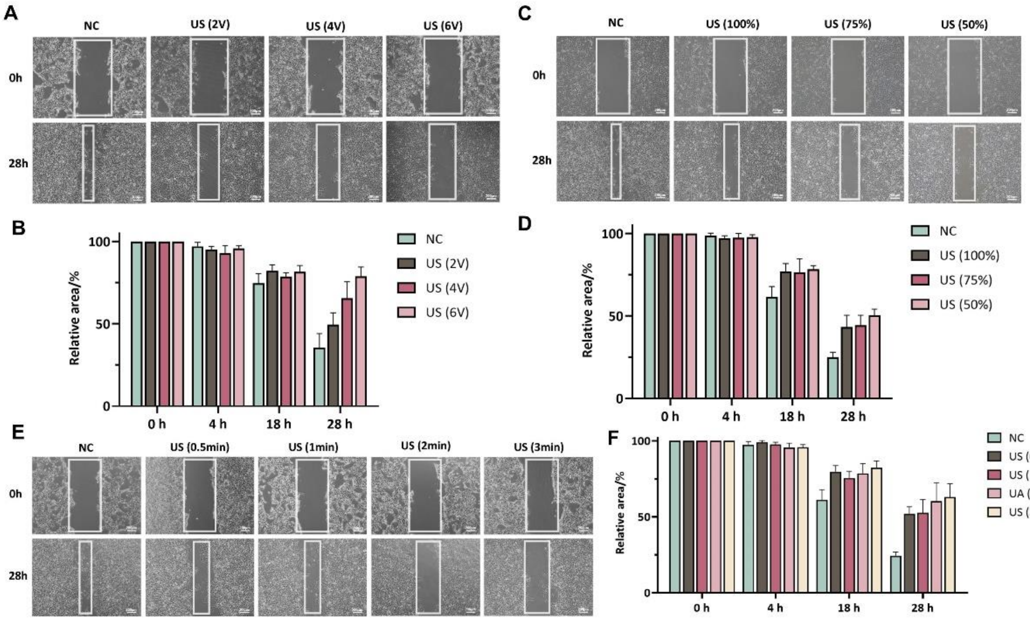

Figure S2. Wound healing and migration assay of NIH 3T3 cells after different LFS. A) Keep the time duration is 3 min and the duty cycle is 100%, changing the voltage of LFS. Randomly selected images of the gap of NIH 3T3 cells at 0 h, 4 h, 18 h, and 28 h after LFS. B) Relative area of the gap in NIH 3T3 over time. C) Keep the time duration is 3 min and voltage is 6 V, changing the duty cycle of LFS. Randomly selected images of the gap of NIH 3T3 cells at 0 h, 4 h, 18 h, and 28 h after LFS. D) Relative area of the gap in NIH 3T3 over time. E) Keep the duty cycle is 100% and voltage is 6 V, changing the time duration of LFS. Randomly selected images of the gap of NIH 3T3 cells at 0 h, 4 h, 18 h, and 28 h after LFS. F) Relative area of the gap in NIH 3T3 over time.

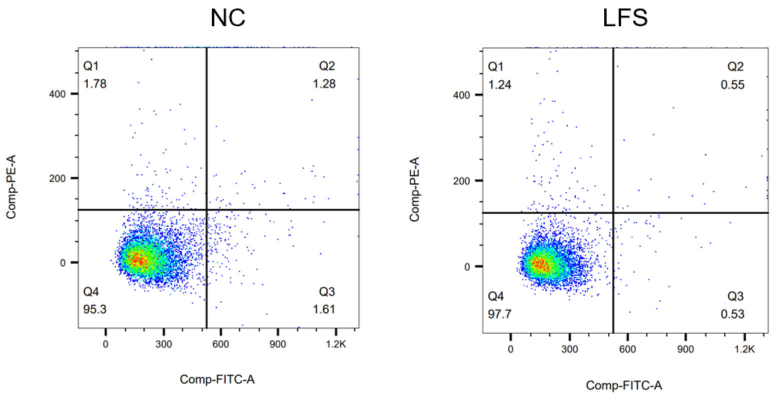

Figure S3. Apoptosis analysis of NIH 3T3 with or without LFS.

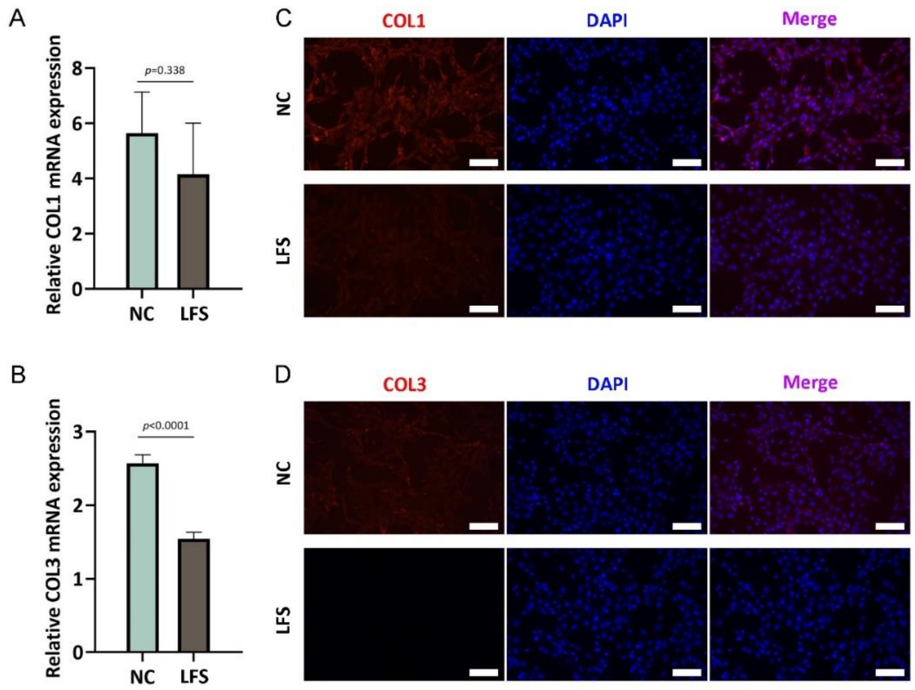

Figure S4. LFS inhibits the synthesis of collagen. A) Relative collagen type I (COL1) mRNA expression of NIH 3T3 cells with or without LFS stimulation. B) Relative collagen type III (COL3) mRNA expression of NIH 3T3 cells with or without LFS stimulation. C) Immunofluorescence staining of COL1 in NIH 3T3 cells with or without LFS stimulation. D) Immunofluorescence staining of COL3 in NIH 3T3 cells with or without LFS stimulation. Scale bar: 100 μm.

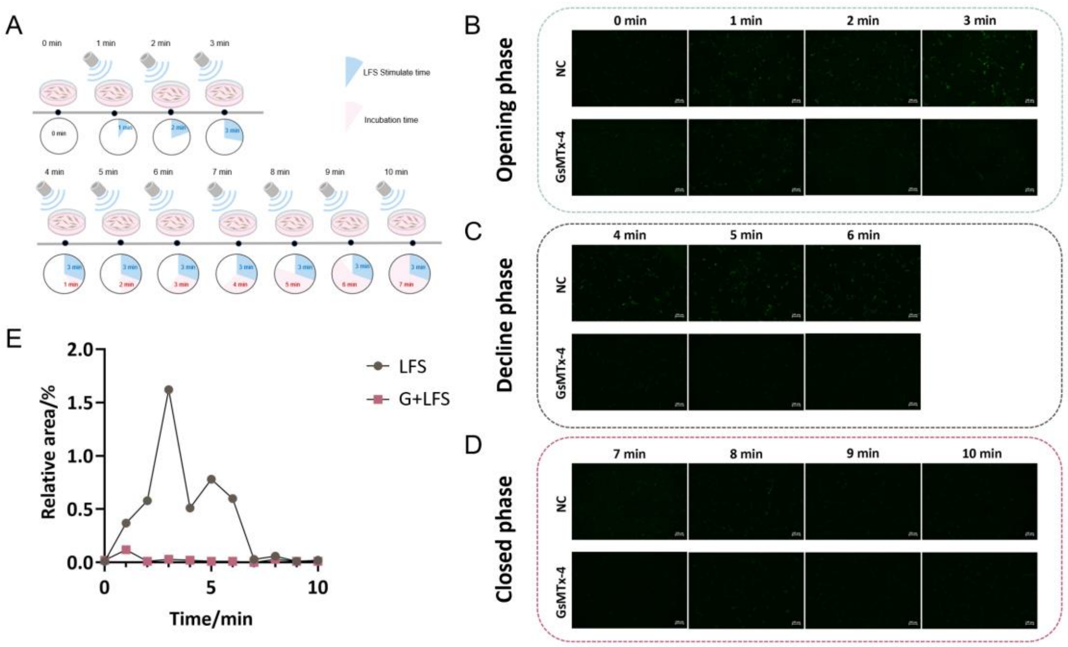

Figure S5. Three phases of LFS-induced calcium influx. A) Schematic diagram showing the intervention schedules. B-D) Calcium levels of PKF cells were labeled by Fluo-8 after different interventions. Three phases were divided according to the fluorescence intensity of intracellular calcium. E) Quantitative visualization of intracellular calcium level.

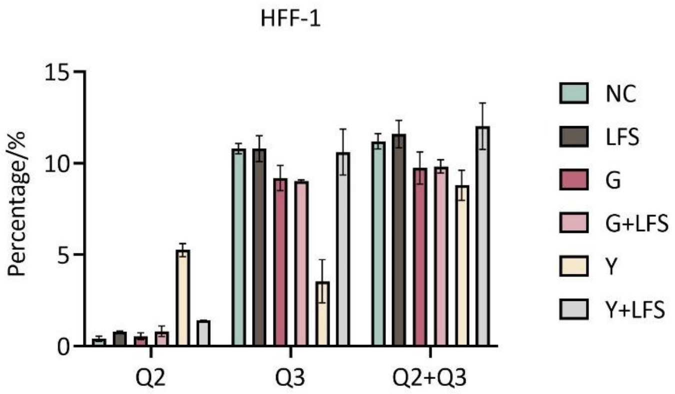

Figure S6. Apoptosis detection of HFF-1 cells. Quantitative analysis of early apoptosis, advanced apoptosis, and total apoptosis of HFF-1 cells. G: GsMTx-4, Y: Yoda1.

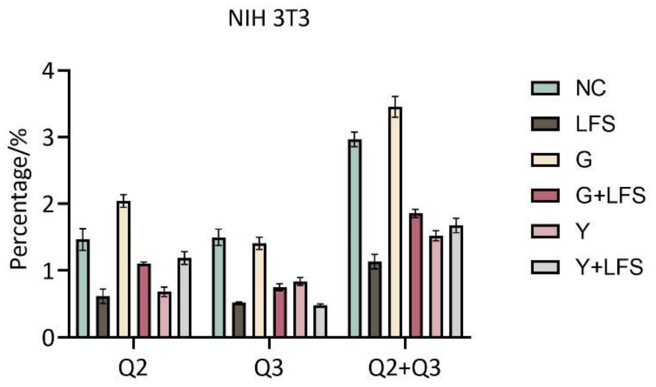

Figure S7. Apoptosis detection of NIH 3T3 cells. Quantitative analysis of early apoptosis, advanced apoptosis, and total apoptosis of NIH 3T3 cells. G: GsMTx-4, Y: Yoda1.

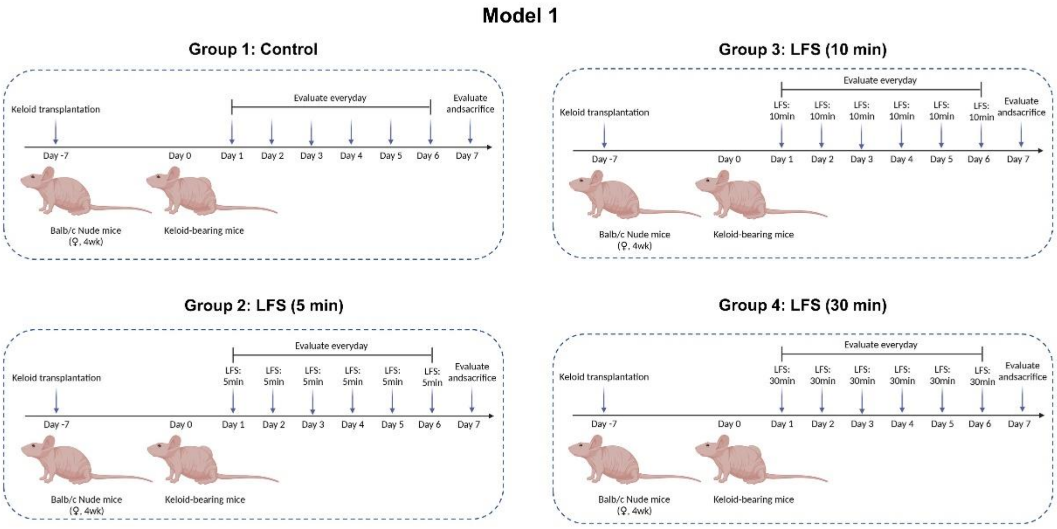

Figure S8. The treatment protocol of Model 1.

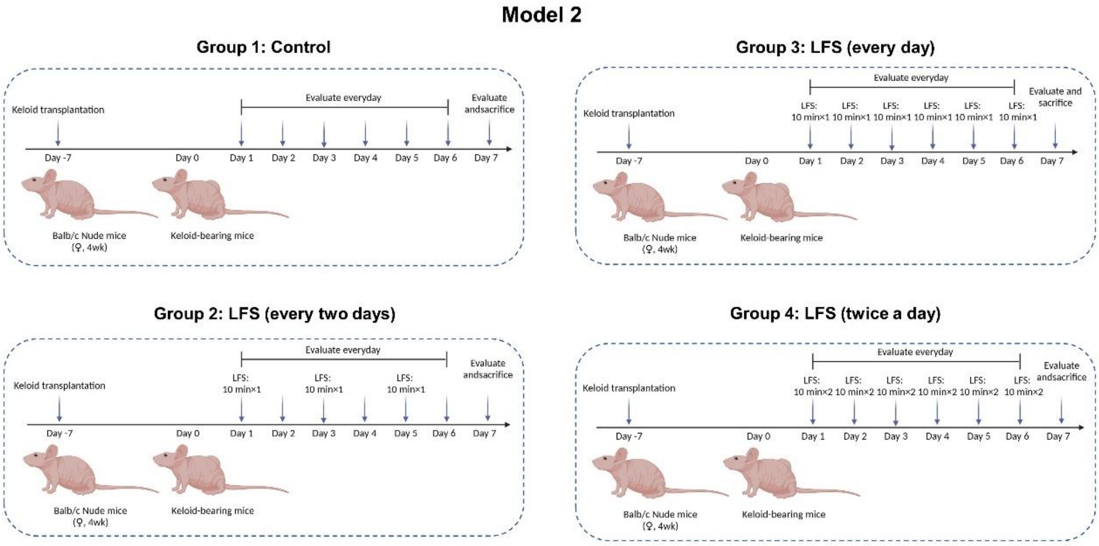

Figure S9. The treatment protocol of Model 2.

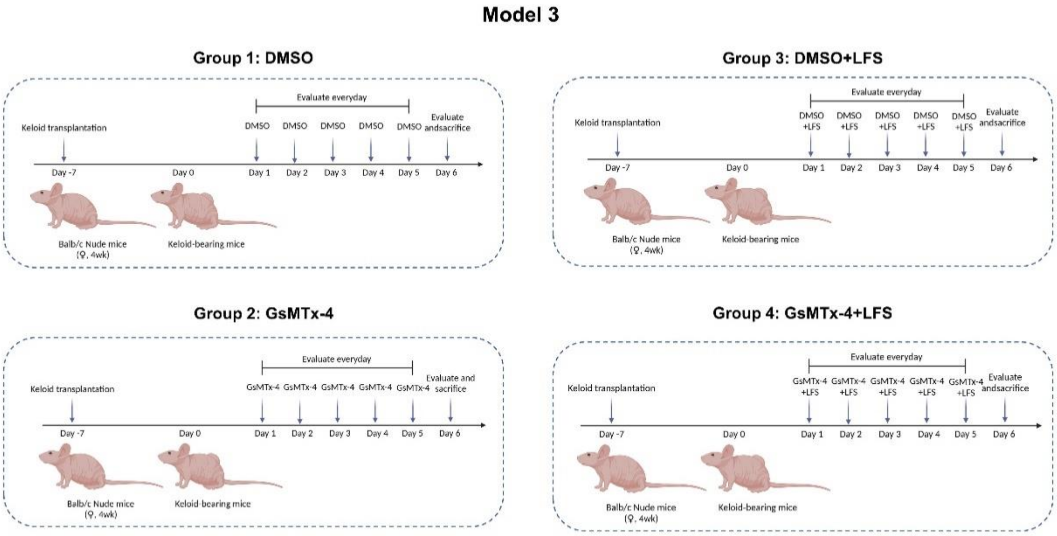

Figure S10. The treatment protocol of Model 3.

Table S1. Published article concerning ultrasound and Piezo1

NO	ID	Supplement of Ultrasound	Effect on Piezo1	Target cells/tissues	Piezo1-dependent effects
1	Bone Res *2021*	None	Activation	Osteoblastic cells (mouse)	Increasing migration and proliferation, and promoting the accumulation of F-actin in the perinuclear region
2	PNAS *2023*	None	Not declared	Brain (mouse)	Inducing neuronal calcium responses, limb movement, and muscle electromyogram responses
3	Ultrason Sonochem *2021*	Piezo1-targeted microbubbles	Activation	neuroblasts (mouse)	Not declared
4	iScience *2022*	Microbubbles	Activation	Pancreatic ductal adenocarcinoma cells (human)<br>Pancreatic ductal adenocarcinoma (xenografts model)	Inducing the apoptosis of PDAC cell lines and tumors
5	Ultrasound Med Biol *2018*	None	Activation	Ovary cells (Chinese Hamster)<br>Embryonic kidney cells (human)	Not declared
6	PNAS *2018*	None	Activation	T cell (human)	Guiding gene activations
7	Research (Wash D C) *2023*	Magnetic field	Activation	Brain (mouse)	Enhancing autophagy to promote the phagocytosis and degradation of β-amyloid through the activation of microglial Piezo1 and alleviated neuroinflammation, synaptic plasticity impairment, and neural oscillation abnormalities.
8	Bioeng Transl Med *2021*	None	Activation	Breast cancer cells (human)	Mediating mechanoptosis
9	J Nanobiotechnology *2023*	2D nanomaterials	Downregulation	Neural stem cell (rat)<br>Spinal cord injury model (mouse)	Promoting neurogenesis and inhibiting inflammation
10	iScience *2019*	None	Activation	Cortical neurons (mouse)<br>Neuronal cells (mouse)	Increasing nuclear c-Fos expression in primary neurons and increasing the expression of the important proteins phospho-CaMKII, phospho-CREB, and c-Fos in a neuronal cells
11	Sci Rep *2021*	None	Activation	HEK293T-P1KO cells (transfected with Mouse Piezo1)	Not declared
12	J Endod *2017*	None	Upregulation	Dental stem cells (rat)	Increasing proliferation

13	APL Bioeng *2023*	None	Activation	Monocytes (human)	Modulating cytokine release
14	Cancers (Basel) *2022*	None	Activation	Macrophage (human) Glioma model (mouse)	Mediates macrophages (M1 and M2) infiltrating
15	Int J Mol Sci *2023*	None	Activation	Motor cortex (rat)	Involving in the cortical inhibitory neuromodulation process
16	Brain Sci *2023*	None	Downregulation	Hippocampus (mice)	Improving cognitive impairment and increasing hippocampal synaptogenesis
17	Biochem Biophys Res Commun *2019*	None	Activation	HEK293T-P1KO cells (transfected with Mouse Piezo1)	Not declared
18	Int J Stem Cells *2022*	None	Activation	Periodontal ligament stem cells (human)	Promoting endothelial differentiation and angiogenesis of periodontal ligament stem cells
